# Tuberculous Spondylodiscitis with Epidural Spread

**DOI:** 10.1590/0037-8682-0617-2022

**Published:** 2023-03-27

**Authors:** Handan Alay, Bahar Yılmaz Çankaya

**Affiliations:** 1 Ataturk University, Faculty of Medicine, Department of Infectious Diseases and Clinical Microbiology, Erzurum, Turkey.; 2 Ataturk University, Faculty of Medicine, Department of Radiology, Erzurum, Turkey.

**FIGURE 1:**
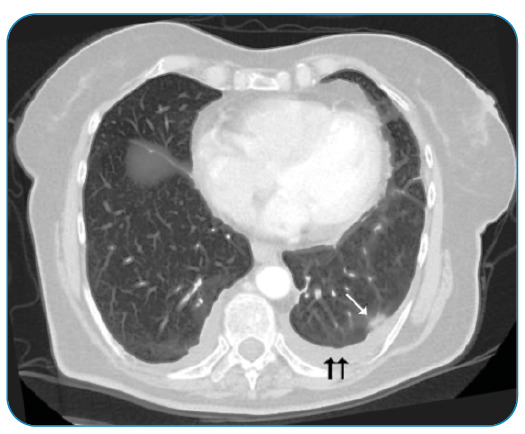
Thoracic computed tomography; Parenchymal nodules, ground-glass appearance (white arrow) and pleural effusion (black arrows) are seen in the lower lobe of the left lung.

**FIGURE 2:**
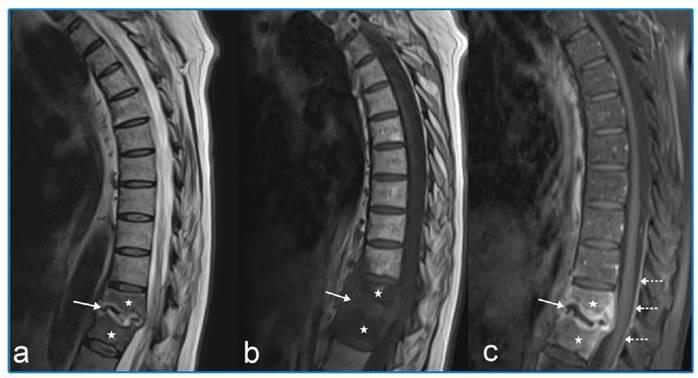
Pre-treatment MRI. Sagittal T2 WI **(a)**: hypointense appearance are seen in the vertebra T11 and T12 (star), irregularity in the disc space, erosions in the vertebral plates, and increased intensity (arrow). T1 WI **(b)**: marked signal losses (arrow) are seen in the vertebral bodies (star) and disc space. Contrast-enhanced T1 WI **(c)**: contrast increases are seen in the vertebral bodies (star), disc space and vertebral plates (arrow), and posterior epidural space (dashed arrow).

**FIGURE 3:**
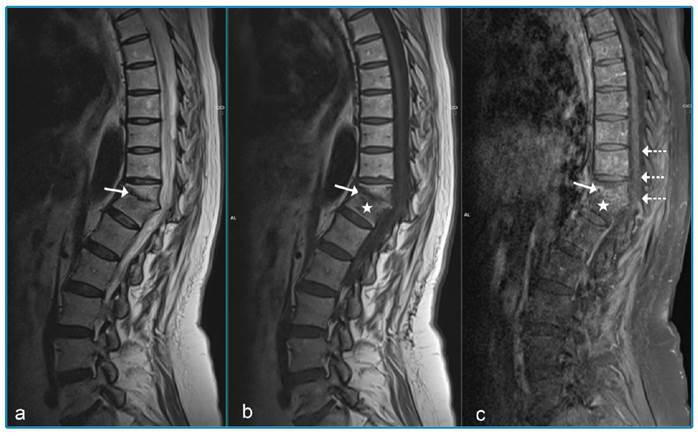
Post-treatment MRI. T2 W **(a):** loss of T11-T12 intervertebral disc space and compression (arrow) in T11 vertebral body is observed. T1 WI **(b):** signal losses in the vertebral bodies and disc region seem to have almost completely recovered. Contrast-enhanced T1 WI **(c):** contrast enhancement is not seen in the vertebral bodies (star) and posterior epidural space (dashed arrow), and the contrast enhancement in the vertebral plates is significantly reduced (arrow).

A 76-year-old foreign-national woman presented with complaints of back pain and weight loss for approximately five months, together with worsened pain, weakness, lack of appetite, and nausea for the preceding two weeks. Restricted vertebral movement and kyphosis were observed during physical examination. The patient’s white cell count was 10.98 × 10^3^/µL, hemoglobin was 11.6 g/dL, C-reactive protein (CRP) was 81.06 mg/L, erythrocyte sedimentation rate (ESR) was 81 mm/h, and tuberculin skin test was 0 mm. Acid-resistant bacilli in sputum were negative. Rose Bengal, Wright agglutination, and Brucella IgM and IgG tests were negative. There was no response to the antibacterial therapy. In the light of pulmonary computed tomography (Figure 1) and thoracic magnetic resonance imaging (MRI) findings (Figure 2), the patient was administered isoniazid (300 mg/day), rifampicin (600 mg/day), ethambutol (2 g/day), and pyrazinamide (2 g/day). On the 15^th^ day of treatment, the CRP decreased to 49.67 mg/L and ESR to 69 mm/h. By the fifth month of treatment, the clinical and laboratory parameters had returned to normal. Thoracic MRI results at that time are shown in Figure 3.

According to a World Health Organization global report, tuberculosis is an increasingly prevalent major cause of morbidity and mortality^1^. Tuberculous spondylodiscitis most frequently affects the thoracolumbar region. The most frequent symptoms are pain, muscle spasms, and associated movement restriction^2^. MRI is quite sensitive in identifying vertebral involvement and epidural extension of the disease^3^. It also assists in differentiating bacterial infections from tuberculous infections and in post-treatment follow up.
